# Mitochondrial Aconitase Enzymatic Activity: A Potential Long-Term Survival Biomarker in the Blood of ALS Patients

**DOI:** 10.3390/jcm12103560

**Published:** 2023-05-19

**Authors:** Cristina González-Mingot, Francisco Javier Miana-Mena, Pedro José Iñarrea, Cristina Iñiguez, José Luis Capablo, Rosario Osta, Anna Gil-Sánchez, Luis Brieva, Pilar Larrodé

**Affiliations:** 1 Neurology-Department, Hospital Arnau de Vilanova of Lleida, 25198 Lleida, Spain; 2 LAGENBIO-Centro de Investigación Biomédica en Red de Enfermedades Neurodegenerativas (CIBERNED), Aragon Institute for Health Research (IIS Aragon), Zaragoza University, 50013 Zaragoza, Spain; 3 Biochemical-Department of Biology-Faculty, Zaragoza University, 50009 Zaragoza, Spain; 4 Neurology-Department, Hospital Clínico Universitario Lozano Blesa of Zaragoza, 50009 Zaragoza, Spain; 5 Neurology-Department, Hospital Universitario Miguel Servet of Zaragoza, 50009 Zaragoza, Spain

**Keywords:** aconitase, ALS, biomarker, enzymatic activity, mitochondrial antioxidant activity

## Abstract

Background: Amyotrophic lateral sclerosis (ALS) is a multisystemic, progressive, neurodegenerative disorder. Despite it being generally fatal within a period of 2–4 years, it is highly heterogeneous; as a result, survival periods may vary greatly among individual patients. Biomarkers can serve as tools for diagnosis, prognosis, indicators of therapeutic response, and future therapeutics. Free-radical-dependent mitochondrial damage is believed to play a crucial role in neurodegeneration in ALS. Mitochondrial aconitase, which is also known as aconitase 2 (Aco2), is a key Krebs cycle enzyme and is involved in the regulation of cellular metabolism and iron homeostasis. Aco2 is very sensitive to oxidative inactivation and can aggregate and accumulate in the mitochondrial matrix, causing mitochondrial dysfunction. Loss of Aco2 activity may therefore reflect increased levels of mitochondrial dysfunction due to oxidative damage and could be relevant to ALS pathogenesis. The aim of our study was to confirm changes in mitochondrial aconitase activity in peripheral blood and to determine whether such changes are dependent on, or independent of, the patient’s condition and to propose the feasibility of using them as possible valid biomarkers to quantify the progression of the disease and as a predictor of individual prognosis in ALS. Methods: We measured the Aco2 enzymatic activity in the platelets of blood samples taken from 22 controls and 26 ALS patients at different stages of disease development. We then correlated antioxidant activity with clinical and prognostic variables. Results: Aco2 activity was significantly lower in the 26 ALS patients than in the 22 controls (*p* < 0.05). Patients with higher levels of Aco2 activity survived longer than those with lower levels (*p* < 0.05). Aco2 activity was also higher in patients with earlier onset (*p* < 0.05) and in those with predominantly upper motor neuron signs. Conclusions: Aco2 activity seems to be an independent factor that could be used in the long-term survival prognosis of ALS. Our findings suggest that blood Aco2 could be a leading candidate for use as a biomarker to improve prognosis. More studies are needed to confirm these results.

## 1. Introduction

Amyotrophic lateral sclerosis (ALS) is a multisystemic neurodegenerative disorder that is characterized by the progressive loss of both cortical and anterior horn spinal motor neurons (MNs). In Europe, ALS is found in approximately 1 of every 50,000 members of the population, and there is a cumulative lifetime risk of its development in 1 of every 400 people [1,2]. Cases of inherited ALS (familial ALS, or FALS) are relatively infrequent (fewer than 10%), with only 20% of these being attributable to a single, specific molecular defect [3]. Although ALS is generally fatal amongst such patients, resulting in death within a period of 2–4 years, survival times vary considerably for individual patients [4]. The etiology of the majority of ALS cases (the remaining 90%, which are classified as sporadic ALS, or SALS) remains unknown, but oxidative stress is generally thought to be involved and to play an important role in causing motor neuron death [5]. Mitochondrial degeneration and protein aggregation mediated by oxidative stress also appear to play major roles in associated progressive motor neuron death [6,7]. Mitochondrial proteins linked to energy production have been identified as key targets for oxidative stress; this is logical given that mitochondria are the main intracellular source of free radicals. In consequence, it is widely believed that free-radical-dependent mitochondrial damage plays a fundamental role in neurodegeneration associated with ALS [8,9,10].

Unfortunately, substantial loss of motor neurons has already occurred by the time the first symptoms appear [11]. Furthermore, the only two drugs approved for use in the treatment of ALS (riluzole and edaravone) have limited beneficial effects on disease progression [12].

Biomarkers can be used as tools for early diagnosis, predictors of prognosis, indicators of target engagement or therapeutic response, and to enable the discovery of future therapeutics for ALS [13,14]. Increasing our knowledge about energy-metabolism-related molecules, including Aco2, may help us to find an efficient therapeutic strategy [15].

In recent decades, several ALS-related biological biomarkers have been recorded, some of which relate to blood [16,17,18,19]. Since we know that this is a multisystemic disease, the identification of a panel of biomarkers that accurately reflect features of its pathology is a priority, not only for diagnostic purposes but also for prognostic and predictive applications [20].

Elevated levels of oxidative stress and altered activity of antioxidant defense enzymes (ADEs) have been found not only in the nervous system but also in the peripheral tissues of FALS and SALS patients [5,6,7,8]. However, it has not yet been established whether oxidative stress is the cause or the consequence of the neurodegenerative process [9].

Mitochondrial aconitase, which is also called aconitase 2 (Aco2), is a key Krebs cycle enzyme and is composed of an iron–sulfur cluster that is highly sensitive to oxidative damage. Aco2 is located in the mitochondrial matrix and is involved in energy generation. It is, however, susceptible to high levels of oxidative stress, which can lead to the inactivation of its activity [21]. Aconitase has been identified as a protein that can undergo oxidative modification and suffer inactivation due to aging and certain oxidative-stress-related disorders. This enzyme is involved in the regulation of cellular metabolism and iron homeostasis, balancing the regulatory, and damaging, effects of reactive oxygen species (ROS) [21]. Studies conducted in animal and cellular models have shown that free radicals and products of oxidative damage can promote the oxidation of Aco2, resulting in impaired enzymatic activity [22]. This can lead to Aco2 aggregating and accumulating in the mitochondrial matrix, causing mitochondrial dysfunction [21]. There is increasing evidence to support a direct association between impaired energy metabolism and the incidence and progression of neurodegenerative disorders in neuronal cells. It has been shown that alterations in bioenergetic parameters are a common pathological feature of neurodegenerative diseases that lead to neuronal dysfunction [15]. 

Loss of Aco2 activity may, therefore, reflect increased levels of mitochondrial dysfunction due to oxidative damage and could be relevant to ALS pathogenesis. 

The aim of our study was to confirm changes in Aco2 activity and to determine whether these changes were dependent on, or independent of, the patient’s condition. We also sought to explore the feasibility of using them as valid biomarkers with which to quantify disease progression and also as individual predictors for prognosis. We analyzed the correlation between mitochondrial antioxidant activity and several variables that are relevant to the clinical evolution of the disease (age, time from onset, bulbar or spinal onset, and UMN or LMN signs) and its severity criteria (nutritional and respiratory statuses), ALS functional rating scores (ALSFRSs) [23,24,25], as well as prognostic factors—such as lipid [26,27] and ferritin levels [28,29]—were studied in a series of ALS patients at different disease stages.

## 2. Materials and Methods

### 2.1. Study Design and Patients

This was a multi-center cohort study that aimed to analyze the potential use of platelet Aco2 activity from isolated mitochondria as a prognostic biomarker. We determined Aco2 activity in 22 controls and in 26 ALS patients at different disease stages in a cross-sectional cohort (some were long-term survivors, while others were in the end-stage of the disease). All the patients were diagnosed by the ALS unit of the Lozano Blesa and Arnau de Vilanova University Hospital as having either definite, probable, or laboratory-supported probable ALS, as defined in a revised version of the El Escorial criteria [30]. None of those studied had a family history of ALS. The exclusion criterium was the presence of other consumptive diseases, such as cancer, systemic infections, and/or autoimmune diseases. The inclusion criteria for the control subjects were as follows: (i) no clinical signs of any neuromotor disorder (e.g., frontotemporal dementia (FTD)), (ii) no family members with ALS, and (iii) no metabolic disorder. The exclusion criteria for controls were as follows: (i) presenting any pathology associated with ALS, such as respiratory diseases, difficulty in swallowing, and cognitive disorders, (ii) having comorbidities, such as diabetes and/or hypertension, (iii) showing signs of suffering any acute and/or chronic inflammatory disorders, and (iv) being a habitual smoker. Control subjects were age- and gender-matched.

This study was conducted according to the ethical principles of the World Medical Association Declaration of Helsinki. Ethical approval was obtained from the Arnau de Vilanova of Lleida Hospital’s ethics committee with approval ID CEIC-2073, and written informed consent was obtained from all the participants. 

### 2.2. Clinical Variables

The clinical variables were recorded paying close attention to variables that were relevant to prognosis (age of onset, bulbar or spinal onset, initial upper or lower MN, and survival) and disease severity (forced vital capacity (FVC < 50%)), body mass index (BMI < 18.5), nutritional status (proteins and Cu), ALSFRS [23,24], triglycerides, cholesterol (total, low-density lipoprotein (LDL), and high-density lipoprotein (HDL)), and ferritin. 

### 2.3. Biochemical Procedures

Platelet-rich plasma from blood samples (50 mL) was extracted and centrifuged. After washing, the platelets were subjected to hypo-osmotic shock, and the mitochondria that were released were isolated via differential centrifugation [31]. Proteins were determined using the Bradford assay [32]; Aco2 activity was analyzed as described by Drampier et al. [33]. 

This technique is based on the fact that digitonin selectively permeabilizes the plasma membrane and leaves the inner mitochondrial membrane intact. Inhibition of mitochondrial aconitase interrupts the flow of citrate through the Krebs cycle. The expression level of Aco2 is expressed in units of activity (mU/mg protein). Aco2 activity was measured in duplicate for each sample and for all samples at the same time. The mean value of two measurements for each sample was used for the analysis. The COX (Complex IV) subunit Vb was used as a mitochondrial protein loading control. 

For each subject, and in all experiments, Aco2 activity was measured in amounts of pelleted purified mitochondria corresponding to 80 µg of mitochondrial proteins. The assessment of Aco2 activity was conducted in 2 mL of a reaction mixture containing 13 mM of Tris (pH 7.4), 5 mM of sodium citrate, 0.2 mM of Beta NADP, 0.6 mM of MnCl_2_, 2 U/mL of isocitrate dehydrogenase, and pelleted purified mitochondria. Aco2 activity was measured spectrophotometrically by following the linear absorbance change at 340 nm, at +37 °C, and at a pH of 7.4 for 30 min.

### 2.4. Statistical Analyses

SPSS 15.0 was used to analyze and compare the mean enzymatic activity levels between groups. Aco2 activity levels in ALS patients were analyzed for correlations with clinical variables. Receiver operating characteristic (ROC) curves were used to calculate the accuracy of the different tests, with the purpose of discerning the ability of the antioxidant activity of aconitase to predict long-term survival [34].

## 3. Results

### 3.1. Demographic and Clinical Variables (Table 1)

There were 26 ALS patients (15 men and 11 women) who were 43–73 years of age (mean 58.24 ± 14.85) and 22 healthy controls (11 men and 11 women) aged 46–69 years (mean 57.60 ± 11.13). There were no significant differences between the groups in terms of sex or age. Four of the ALS cases had the bulbar onset form of the disease, while the rest had the spinal onset form. Despite the fact that all the patients had UMN and LMN involvement at the time of the study (they met The El Escorial criteria for definite, probable, or laboratory-supported probable ALS), in the beginning, UMN involvement was predominant in six cases, while LMN involvement was more prominent in the rest. The ALS patients were at different stages of disease development; some were long-term survivors, while others were in the end-stage of the disease.

Regarding disease severity, 10 subjects had FVCs of <50%, and 2 had BMIs of <20 (but none had a BMI of <18) at the moment of antioxidant activity determination. The ALSFRS scores ranged from 1 to 32 points and were grouped according to severity (high: ALSFRS of 1–12; medium: ALSFRS of 13–20; and mild: ALSFRS of 21–32). The time that had elapsed since the onset of the disease ranged between 6 and 203 months. There were 14 patients with longer-term survival (>36 months), 6 of whom survived for >48 months, while 22 patients had died by the time the study was performed (6 of them died within 12 months of antioxidant activity determination). 

Regarding the other analytical parameters, 3 ALS patients had triglyceride values of >150 mg/dL, 10 had total cholesterol levels of >200 mg/dL (6 had total cholesterol levels of >240 mg/dL), 9 had low-density lipoprotein (LDL) levels of >130 mg/dL (6 had LDL levels of >160 mg/dL), and 4 had high-density lipoprotein (HDL) levels of <35 mg/dL, while 2 male ALS patients had ferritin values of >300 ng/mL and 4 female patients had ferritin levels of > 150 ng/mL at the moment when antioxidant activity was determined.

### 3.2. Aco2 Activity in ALS vs. Controls

**Figure 1:**
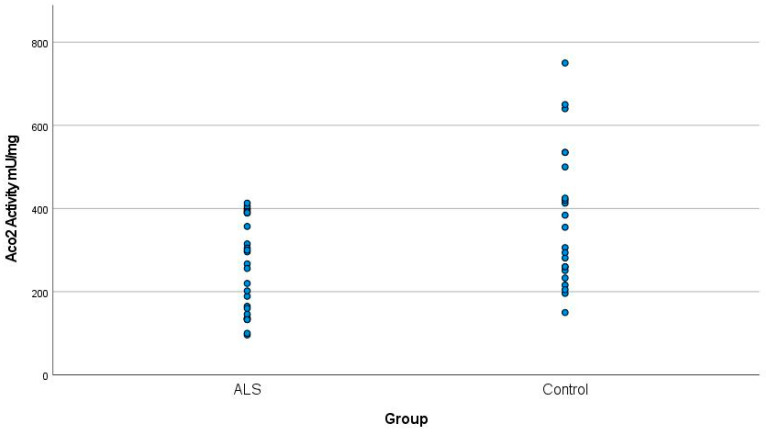
Mean Aco2 activity in ALS patients and controls. The value of Aco2 activity in the ALS patients was 257.65 ± 111.1 mU/mg, whereas in the controls, it was 375.41 ± 166 mU/mg (*p* < 0.05). ALS: Amyothophic lateral sclerosis. Aco2: Aconitase 2.

Aco2 activity was significantly lower in the 26 ALS patients than in the 22 healthy controls: the value range was 146.55 to 368.75 mU/mg in ALS patients versus 209.41 to 541.41 mU/mg in controls (Student’s *t* = −2.9; *p* < 0.05) (Figure 1). These results were independent of age. 

### 3.3. Aco2 Activity and ALS Severity

The patients who died later had the highest Aco2 activity levels; the level of Aco2 activity in the patients who died within 12 months of the determination time was 226.56 ± 94.1 mU/mg, while that in those who were still alive (survival > 48 months) was 326 ± 45.4 mU/mg (*p* < 0.05).

**Figure 2:**
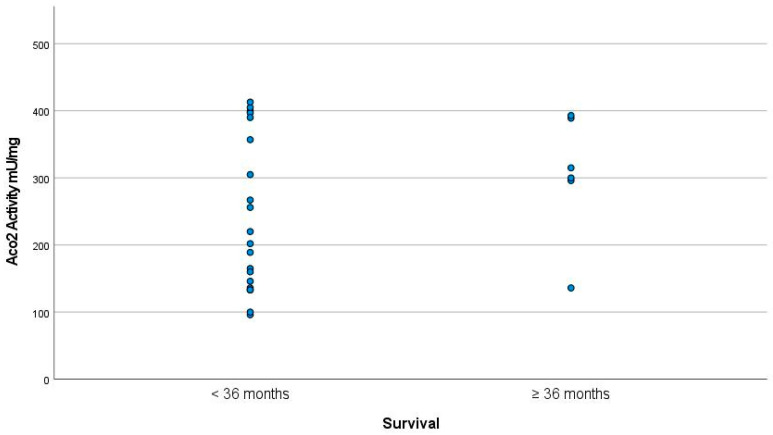
Aco2 activity in subjects with survival periods of ≥36 months was 328.25 ± 90 mU/mg as opposed to 22.28 ± 106.9 mU/mg in subjects with survival periods of <36 months (*p* < 0.05).

The patients with the longest survival periods also had the highest levels of Aco2 activity; the Aco2 activity level was 328.25 ± 90 mU/mg in subjects with survival times of ≥36 months and 226.28 ± 106.9 MU/mg in those with survival times of <36 months (*p* < 0.05) (Figure 2).

When we divided the sample into two groups based on very-long-term survival, we found that 6 patients lived for >48 months, as opposed to 20 who lived for <48 months. When comparing the Aco2 activity of each group with the control group, we found that the Aco2 activity in the group of patients with survival times of >48 months was 304.83 ± 93.35 mU/mg vs. 374.41 ± 166.01 mU/mg in controls (*p* = 0.61). Meanwhile, the Aco2 activity in the group of patients with survival times of <48 months was 243.5 ± 114.23 mU/mg vs. 374.41 ± 166.01 mU/mg in controls (*p* < 0.005). 

**Figure 3:**
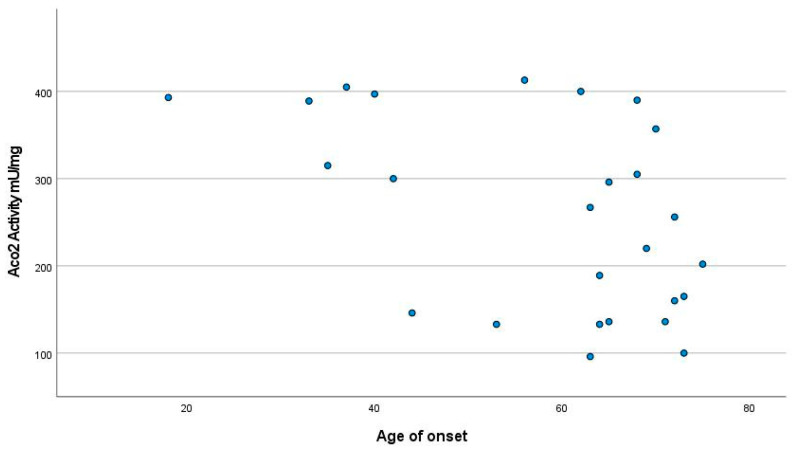
Aco2 activity levels were higher in patients with early onset than in the late group (*p* < 0.01).

**Figure 4:**
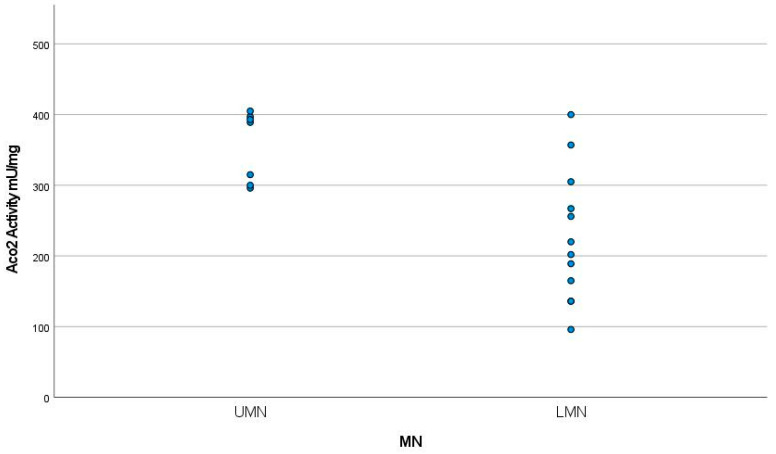
Aco2 activity levels were higher in patients with UMN than in those with predominantly LMN involvement (Aco2 activity of 368.83 ± 47.4 mU/mg versus 227.42 ± 92.9 mU/mg (*p* < 0.05)). UMN: Upper motor neuron, LMN: Lower motor neuron, MN: Motor neuron.

The patients with the onset of the disease at the youngest ages showed the highest levels of Aco2 activity (*p* < 0.01) (Figure 3). Aco2 activity levels were also higher in patients with UMN than in those with predominantly LMN involvement (aconitase activity of 365.83 ± 47.4 mU/mg versus 227.42 ± 92.9 mU/mg; *p* < 0.05) (Figure 4). 

No significant associations were observed between aconitase mitochondrial antioxidant activity and BMI, FVC, lipids, and ferritin levels at the determination time. This could have been because these parameters depend on the condition of each patient and the severity of their disease. In addition, we did not find any statistically significant relationship between ferritin levels and survival (*p* = 0.26) or between ferritin and the ALSFRS-r score in our sample (*p* = 0.3).

### 3.4. ROC Analysis

ROC analysis tests play a fundamental role in assessing the accuracy of tests used to predict events over time; they reveal the prognostic values of specific markers [34]. Based on the results obtained by analyzing the antioxidant activity of Aco2 and its relationship with disease survival, it was decided to proceed with ROC analysis tests. This was conducted with the aim of discerning the ability of the antioxidant activity of Aco2 to predict long-term survival and to demonstrate its prognostic value.

**Figure 5:**
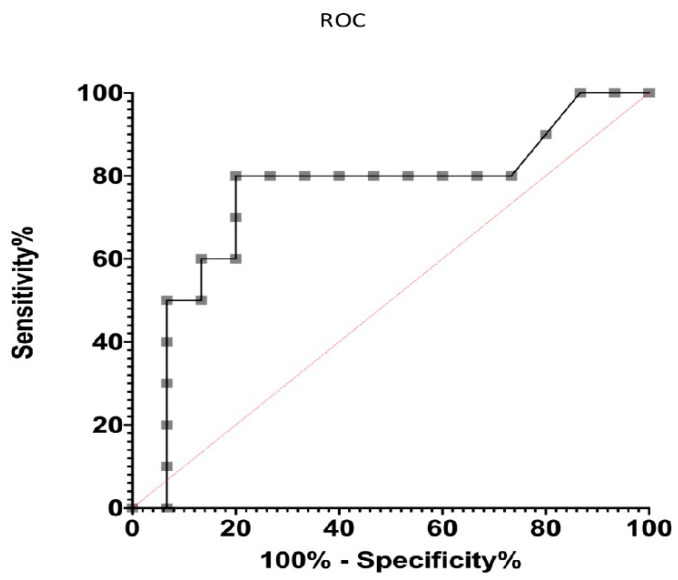
The ROC (receiver operating characteristic) curve is a graphical representation of the performance of a binary classifier that predicts the probability of a positive outcome. The area under the curve (AUC) represents the overall performance of the classifier, with a higher AUC indicating better performance. An AUC of 1.0 indicates perfect classification, while an AUC of 0.5 (red line) indicates random guessing. In our case, Aco2 activity could be considered a good predictor of long-term survival because its AUC value was 0.75 (*p* = 0.03).

In the analysis, it could be observed that Aco2 activity proved to be a good predictor of long-term survival (evolution over 48 months), with AUC (area under the curve) values of 0.75 and *p* = 0.03 (Figure 5).

## 4. Discussion

The present study analyzed Aco2 activity in patients at different stages of ALS severity and compared this with controls. Significant decreases in Aco2 activity were observed in mitochondria obtained from the platelets of ALS patients compared with those in the platelets of controls. We measured mitochondrial antioxidant activity in platelets obtained from peripheral blood since this was easily accessible and was a source of biomarkers for neuroaxonal and muscle degeneration. It may, therefore, be more representative of ALS pathobiology than samples extracted from other sources [35]. We also observed that this decrease in Aco2 activity was independent of our patients’ respiratory, nutritional, and functional statuses. At the onset of the disease, the younger patients in our study exhibited higher levels of Aco2 activity. It has been reported that young patients present UMN involvement more frequently [36]. Advanced age at onset has also been shown to be a predictor of poor prognosis [37,38]. In our study, the activity of this enzyme was high in long-term survivors and in the longstanding phenotype (characterized by early onset and a predominance of upper MN signs). According to our results, higher levels of ACO2 activity were related to better prognoses. Some authors have also suggested a relationship between the progression rate of ALS and reduced antioxidant activity [39,40]. We also observed that patients who had lower levels of Aco2 activity tended to die within 12 months of determination, independent of other parameters. We can therefore predict that patients with lower levels of Aco2 will have shorter survival periods. We did not, however, find any correlations between either lipids or ferritin and Aco2 antioxidant status. This is consistent with the thesis that defective Aco2 activity is also independent of these prognostic factors. 

There is major interest in developing descriptions of diagnostic and prognostic markers in ALS; this would permit early intervention with disease-modifying therapies and also provide information about patient responses [41]. Babu et al. noted that ALS patients with extremely low erythrocyte GSH levels died earlier than others [8]. Over the past 10 years, considerable progress has been made in the characterization of neurofilament light chain (NFL), such as in cerebrospinal fluid (CSF), and blood biomarkers [42]. NFL indicates neuroaxonal injury, independent of causal pathways. Previous studies have shown that elevated levels of NFL [42] and phosphorylated neurofilament heavy chain (pNFH) in plasma, serum, and cerebrospinal fluid appear to be associated with accelerated disease progression in ALS [43,44]. NFL values were negatively correlated with disease durations and ALSFRS-r scores and positively correlated with disease progression rates [43]. Furthermore, Nardo et al. described a biomarker combination of ERp57, CypA, and TDP-43 in peripheral blood mononuclear cells that were able to discriminate between patients with high and low disease severity [45]. Despite TDP-43 playing a key role in the pathogenesis of ALS, attempts to measure disease-specific forms of TDP-43 in peripheral biofluids in ALS patients have yet to yield consistent results [46]. Previous studies have pointed to focal iron accumulation associated with brain iron dyshomeostasis as a hallmark of neurodegeneration [47]. High serum ferritin has been described as an indicator of poor prognosis in ALS [48,49]. Iron homeostasis alterations could trigger susceptibility to an iron-dependent cell-death pathway [48]. Moreover, along the same line, recent studies suggest brain hemoglobin is involved in neurodegenerative processes [50]. However, in our study, we did not find any statistically significant relationship between ferritin levels and survival, or between ferritin levels and ALSFRS-r scale scores. This could, however, have been due to the limited size of our sample.

To the best of our knowledge, no previous works have studied Aco2 activity in the peripheral blood of ALS patients. However, Aco2 activity has been studied in peripheral blood in other neurodegenerative diseases. For example, Aco2 activity was lower in the blood of Huntington’s disease (HD) patients than in controls. Moreover, Aco2 activity correlated significantly with the motor score, functional capacity, and disease duration in HD [51,52]. Aco2 activity has previously been studied in the peripheral blood of subjects with Alzheimer’s disease (AD) and mild cognitive impairment (MCI) [53]. Lower levels of Aco2 activity have been reported in the peripheral blood of patients with AD and MCI than in controls. For this reason, Aco2 has been proposed as a potential candidate for use in monitoring disease progression. However, in these studies, the ACO2 activity levels in the controls were lower than in our study (117 ± 40 mU/mg and 123 ± 40 mU/mgr). This could have been due to the fact that the activity was analyzed in mitochondria extracted from lymphocytes instead of platelets.

Our study did, however, have certain limitations. Our sample size was limited, and despite the cases being consecutively enrolled, a certain selection bias was possible. Factors such as diet and physical activity, which can alter oxidative stress levels, were not controlled, and no blood samples were taken after nightly fasting. It would be beneficial to increase the sample size for future studies and to also compare other biomarkers. It would also be interesting to carry out longitudinal measurements throughout each patient’s disease evolution.

Based on our results, Aco2 activity would seem to be a valid prognostic biomarker; we have not, however, shown that it could be used as a diagnostic tool. Aco2 activity levels in the very-long-term survival group (>48 months) were lower than in the controls, but these differences did not reach levels of statistical significance. Meanwhile, Aco2 activity levels in the short-term survival group (<48 months) were significantly lower than in the controls. This could have been due to the small size of our sample, or it could also suggest that Aco2 activity would not make a useful diagnostic biomarker.

Studying the ROC curves confirmed that Aco2 activity was a good predictor of long-term evolution in ALS. High levels of aconitase could therefore be considered indicative of a longer disease evolution regardless of its severity.

The ROC curve plays a fundamental role in proving the prognostic value of a parameter. A more accurate evaluation of the diagnostic tests could be achieved by extending their sensitivity and specificity. For tests with continuous or ordinal outcomes, the ROC curve provides the best indication of diagnostic accuracy and also constitutes a unifying approach within the test evaluation process [54]. Based on the results obtained, we studied the ROC curves with the aim of discerning the ability to predict the long-term survival of antioxidant activity within Aco2 activity.

ALS is increasingly perceived as a multisystem neurodegenerative disorder; thus, the development of biomarker panels, and multimodal biomarkers, in which different modalities of investigation are combined to obtain more sensitive measurements, will be needed to fully capture all the different facets of ALS pathology [14].

## 5. Conclusions

Aco2 activity seems to be an independent factor that could be used in the long-term survival prognosis of ALS. Our findings suggest that blood Aco2 could be a leading candidate for use as a biomarker to improve prognosis. More studies are needed to confirm these results.

## Figures and Tables

**Table 1:** Description of the sample: demographic and clinical variables and Aco2 activity. Our sample consisted of 26 ALS patients (15 men and 11 women) and 22 healthy controls who were matched for age and sex. Four of the ALS cases displayed the characteristics of bulbar onset ALS, while twenty-two displayed signs of the spinal onset variety. The ALS patients were at different stages of disease development and exhibited different degrees of severity. ALS: Amyothophic lateral sclerosis, UMN: Upper motor neuron, LMN: Lower motor neuron, VCM: Vital capacity maneuver, BMI: Body mass index, Aco 2: Aconitase 2.

	ALS	Control
Sex (male/female)	15/11	11/11
Age (years)	58.24 ± 14.85	57.60 ± 11.13
Riluzole	26	0
Survival > 36 months	14	-
Survival > 48 months	6	-
Site of onset (bulbar/spinal)	4/22	-
UMN/LMN at onset	6/20	-
VCM < 50%	10	-
BMI < 20	2	-
ALSFRS-r High Medium Mild	6 11 9	-
El Escorial criteria Definite Probable Laboratory-supported probable	13 10 3	
Aco2 activity	257.65 ± 111.1 mU/mg	375.41 ± 166 mU/mg

## Data Availability

The data generated during this study are available from the corresponding author upon reasonable request.

## Abbreviations

ALS, amyotrophic lateral sclerosis; Aco2, aconitase 2; FALS, familial ALS; SALS, sporadic ALS; ALSFRS, ALS functional rating score; FVC, forced vital capacity; BMI, body mass index; MN, motor neuron; ROS, reactive oxygen species; ROC, receiver operating characteristics; LMN, lower motor neuron; and UMN, upper motor neuron.
